# A contribution to the study of the Lower Volga center of scarab beetle diversity in Russia: checklist of the tribe Aphodiini (Coleoptera, Scarabaeidae) of Dosang environs

**DOI:** 10.3897/BDJ.1.e979

**Published:** 2013-09-16

**Authors:** Andrey Frolov, Lilia Akhmetova

**Affiliations:** † Zoological Institute of Russian Academy of Sciences, Sankt-Petersburg, Russia

**Keywords:** Scarab beetles, dung-beetles, aphodiines, Caspian lowland desert, faunistic composition

## Abstract

The field sampling of the Aphodiini scarab beetles in Dosang environs (Astrakhan Province, European Russia) in 2006–2012 resulted in the collection of 44 species. All but one of them belong to *Aphodius* Hellwig (*sensu lato*). This is apparently the richest recorded local Aphodiini fauna in Russia. The high Aphodiini diversity in the area can be explained by the long vegetative season with high effective heat sum, large livestock providing abundant food resources throughout the year, and location in the transition belt between Volga-Akhtuba Floodplain and Desert floristic districts. The core fauna consists of mesophilous species widely distributed in the Palearctic region and confined to the intrazonal habitats. Other species have ranges mostly limited to the steppe, semidesert, and desert zones.

## Introduction

The tribe Aphodiini is the most diverse and widespread among the scarab beetles of the Northern Palearctic, including Russia. The members of this group dominate dung beetle communities and a few species can be found as far north as 65 degree of the northern latitude. However, like the majority of other scarabs, Aphodiini are the most diverse in the southern regions of the country. In Russia, major centers of aphodiine diversity are chiefly found in mountainous regions — Caucasus, Altai Mountains, Southern Primorye (Akhmetova 2011). The exception to this is the Caspian lowland desert with rich scarab beetle fauna (Nikolajev 1987, Shokhin 2007). Lower Volga, and especially Caspian lowland desert has long been recognized as a center of scarab beetle diversity in Russia. A number of first country records of aphodiines have originated from this area (Akhmetova and Frolov 2008b, Akhmetova and Frolov 2008a, Akhmetova and Frolov 2009) yet its fauna is still inadequately explored.

We have summarized the data of multi-year survey in Astrakhan Province and provided a check-list of the tribe Aphodiini of the Dosang environs. The list includes 44 species for which all but one of which belong to the mega-diverse genus *Aphodius* Hellwig (= *Aphodius* Illiger) (*sensu lato*). Notes on the distribution of the species and feeding preferences, if known, are given. The biogeographic composition of the fauna and possible reasons of high local species richness are discussed.

## Materials and methods

### Study area

The study area is situated оn the Akhtuba River (fork of Volga River) about 90 km upstream of Volga estuary, Astrakhan Province, European Russia. We were based in Dosang Railway Station (N 46°54' E 47°55'). All species listed below and the majority of specimens were collected within 1 km from the station i.e. within an area of about 3 km^2^.

**Figure 1.**
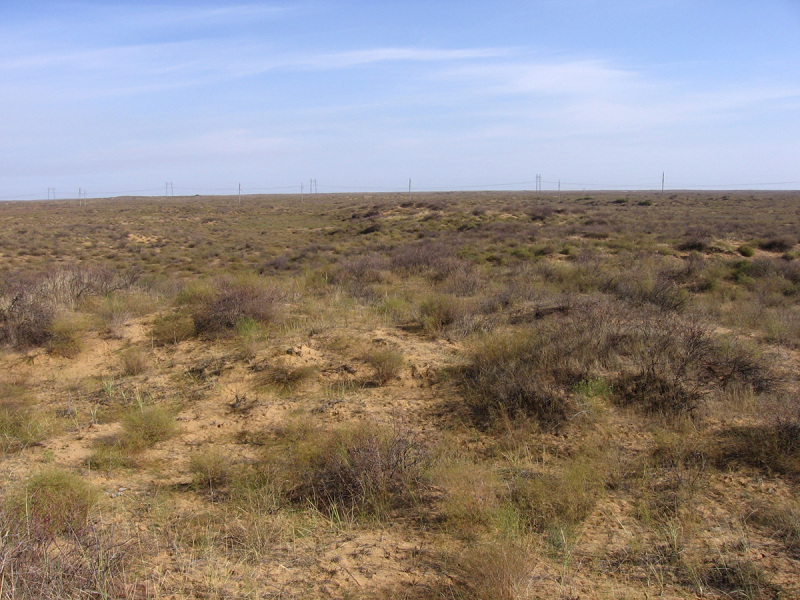
Fixed sands near Dosang, Astrakhan Province, Russia.

**Figure 2.**
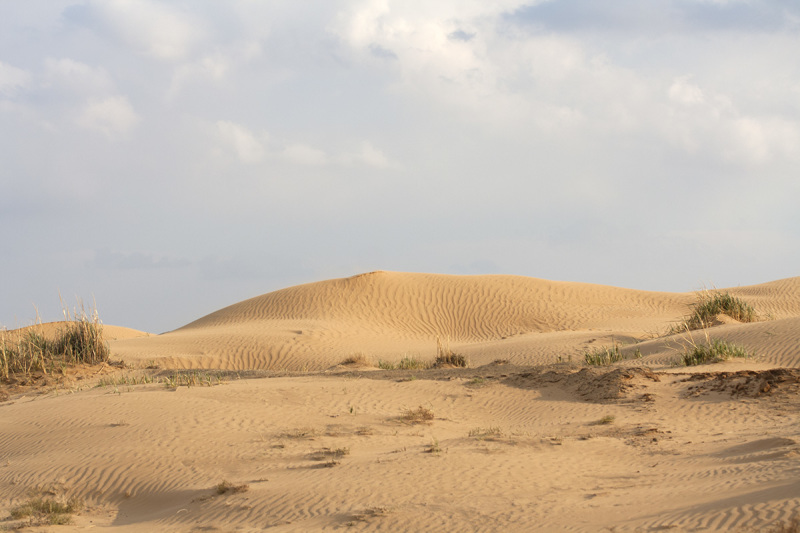
Barchan sands North-East of Dosang, Astrakhan Province, Russia.

**Figure 3.**
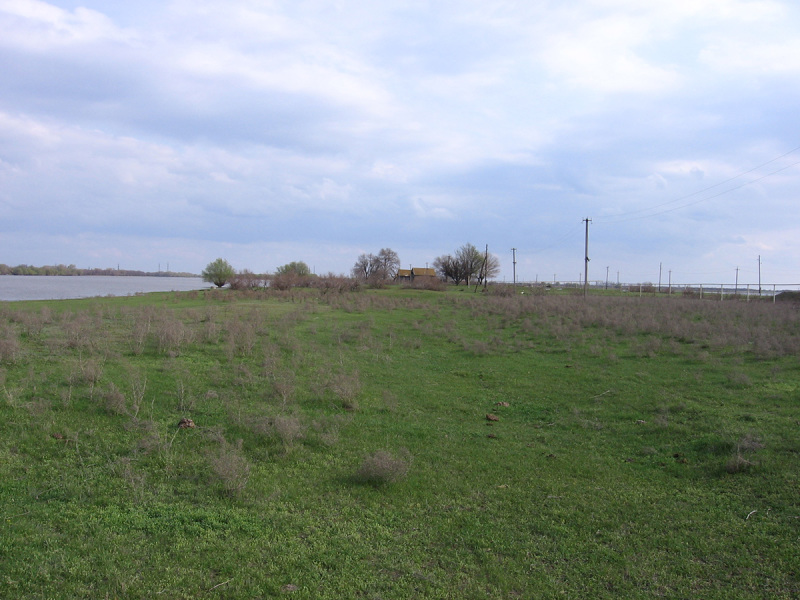
Right side of Akhtuba River, floodplain, Dosang environs, Astrakhan Province, Russia.

**Figure 4.**
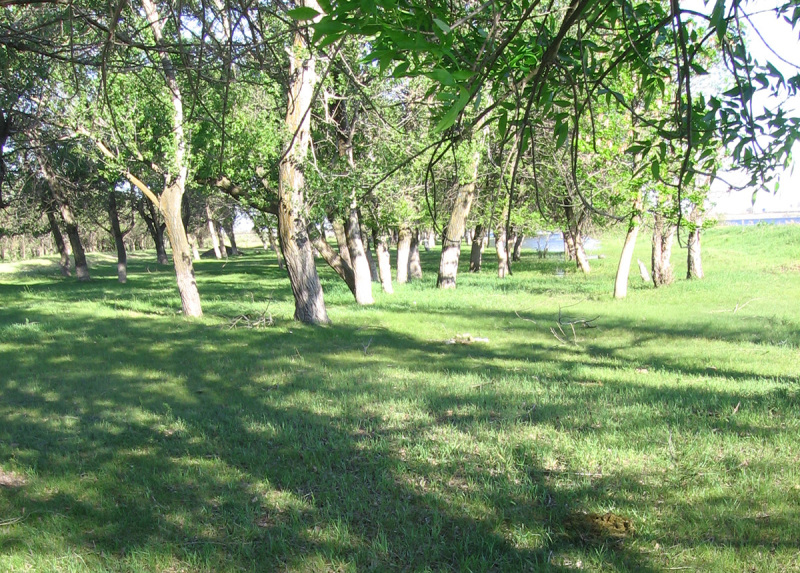
Left side of Akhtuba River, riverine secondary forest near Dosang, Astrakhan Province, Russia.

Four main biotopes were sampled: fixed sands (Fig. 1), barchan sands (Fig. 2), floodplain of Akhtuba River (Fig. 3), and riverside secondary forest (mainly of ash) (Fig. 4).

### Material

The material used in this work was collected by the authors from 2006 to 2012 in different seasons but primarily in spring and fall. Additional specimens were received from colleagues listed in the acknowledgement section. All the material mentioned is deposited in the collection of Zoological Institute of Russian Academy of Sciences, Sankt-Petersburg (ZIN).

### Methods

The beetles were collected by the following methods: washing of fresh horse and cow dung, hand collecting from fresh and old dung, mercury lamp traps, soil sifting with a sieve, hand collecting from marmot holes. The larvae were collected from old dung and soil and some specimens from each lot were reared to adults in the laboratory.

Classification and nomenclature of taxa follow Nikolajev (1987), Kabakov and Frolov (1996), Frolov (2002) and Akhmetova and Frolov (2008b). Classification of distribution ranges of Aphodiini species follows Akhmetova (2011) and is based on the zoogeographical divisions of the Palearctic Region (Emeljanov 1974). *Aphodius* species are arranged alphabetically by epithet.

## Checklists

### Checklist of the tribe Aphodiini of Dosang environs

#### Aphodius aequalis

A. Schmidt, 1907

##### Materials

- **Type status:** Other material. **Occurrence:** recordedBy: A. V. Frolov, L. A. Akhmetova; individualCount: 10; **Location:** country: Russia; stateProvince: Astrakhan'; locality: Dosang Railway Station; decimalLatitude: 46.90; decimalLongitude: 47.92; **Event:** samplingProtocol: light trap; eventDate: 2007-05-23; **Record Level:** collectionID: urn:lsid:biocol.org:col:34969; institutionCode: ZIN; collectionCode: Coleoptera
- **Type status:** Other material. **Occurrence:** recordedBy: A. V. Frolov, L. A. Akhmetova; individualCount: 14; **Location:** country: Russia; stateProvince: Astrakhan'; locality: Dosang environs, fixed sands; decimalLatitude: 46.92; decimalLongitude: 47.92; **Event:** samplingProtocol: cow dung washing; eventDate: 2012-05-15; **Record Level:** collectionID: urn:lsid:biocol.org:col:34969; institutionCode: ZIN; collectionCode: Coleoptera

##### Ecological interactions

###### Feeds on

Cattle dung, mostly cow dung.

##### Distribution

Southern Palearctic region up to Saur-Tarbagataj in the north-east.

#### Aphodius badenkoi

Nikolajev, 1987

##### Materials

- **Type status:** Other material. **Occurrence:** recordedBy: A. V. Frolov, L. A. Akhmetova; individualCount: 1; **Location:** country: Russia; stateProvince: Astrakhan'; locality: Dosang environs, fixed sands; decimalLatitude: 46.92; decimalLongitude: 47.92; **Event:** samplingProtocol: horse dung washing; eventDate: 2007-04-17; **Record Level:** collectionID: urn:lsid:biocol.org:col:34969; institutionCode: ZIN; collectionCode: Coleoptera

##### Ecological interactions

###### Feeds on

Cattle dung.

##### Distribution

Middle Asian deserts, Caspian lowland desert.

#### Aphodius bimaculatus

(Laxmann, 1770)

##### Materials

- **Type status:** Other material. **Occurrence:** recordedBy: A. V. Frolov, L. A. Akhmetova; individualCount: 2; **Location:** country: Russia; stateProvince: Astrakhan'; locality: Dosang environs, fixed sands; decimalLatitude: 46.92; decimalLongitude: 47.92; **Event:** samplingProtocol: horse dung washing; eventDate: 2007-04-17; **Record Level:** collectionID: urn:lsid:biocol.org:col:34969; institutionCode: ZIN; collectionCode: Coleoptera
- **Type status:** Other material. **Occurrence:** recordedBy: A. V. Frolov, L. A. Akhmetova; individualCount: 6; **Location:** country: Russia; stateProvince: Astrakhan'; locality: Dosang environs, fixed sands; **Event:** samplingProtocol: horse dung washing; eventDate: 2007-04-17; **Record Level:** collectionID: urn:lsid:biocol.org:col:34969; institutionCode: ZIN; collectionCode: Coleoptera
- **Type status:** Other material. **Occurrence:** recordedBy: A. V. Frolov, L. A. Akhmetova; individualCount: 1; **Location:** country: Russia; stateProvince: Astrakhan'; locality: Dosang environs, fixed sands; decimalLatitude: 0.00; **Event:** samplingProtocol: horse dung washing; eventDate: 2008-04-04; **Record Level:** collectionID: urn:lsid:biocol.org:col:34969; institutionCode: ZIN; collectionCode: Coleoptera
- **Type status:** Other material. **Occurrence:** recordedBy: A. V. Frolov, L. A. Akhmetova; individualCount: 2; **Location:** country: Russia; stateProvince: Astrakhan'; locality: Dosang environs, left bank of Akhtuba River, floodplain; decimalLatitude: 46.91; decimalLongitude: 47.91; **Event:** samplingProtocol: horse dung washing; eventDate: 2008-04-06; **Record Level:** collectionID: urn:lsid:biocol.org:col:34969; institutionCode: ZIN; collectionCode: Coleoptera

##### Ecological interactions

###### Feeds on

**Figure 5.**
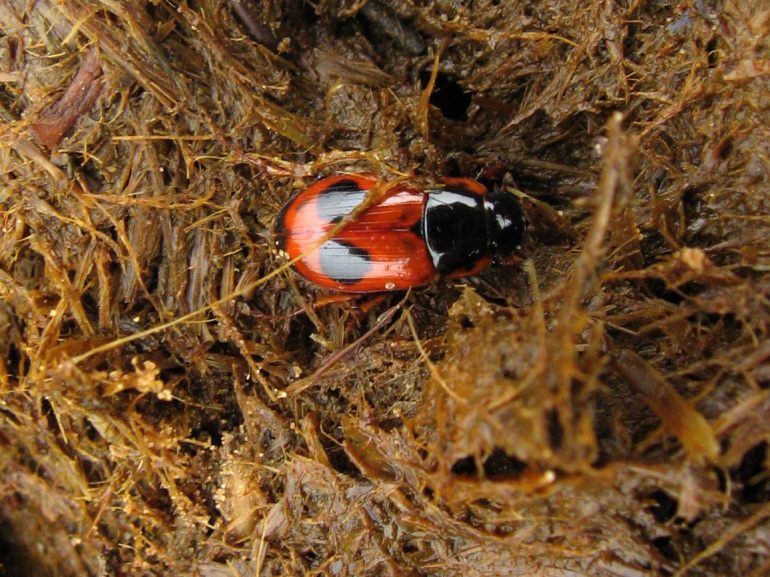
*Aphodius bimaculatus*. Dosang environs, Astrakhan Province, Russia.

Adults and larvae feed on horse dung (Fig. 5).

##### Distribution

Central and Eastern Europe, Western Asia up to East Kazakhstan in the east. The species is mostly occur in forest-steppe and steppe zones.

#### Aphodius caspius

Ménétriés, 1832

##### Materials

- **Type status:** Other material. **Occurrence:** recordedBy: A. V. Frolov, L. A. Akhmetova; individualCount: 1; **Location:** country: Russia; stateProvince: Astrakhan'; locality: Dosang environs, left bank of Akhtuba River, floodplain; decimalLatitude: 46.91; decimalLongitude: 47.91; **Event:** samplingProtocol: horse dung washing; eventDate: 2006-10-06; **Record Level:** collectionID: urn:lsid:biocol.org:col:34969; institutionCode: ZIN; collectionCode: Coleoptera

##### Ecological interactions

###### Feeds on

Cattle dung.

##### Distribution

Steppe zone from Caucasus to West Siberia.

#### Aphodius clathratus

Reitter, 1892

##### Materials

- **Type status:** Other material. **Occurrence:** recordedBy: V. Kozlov; individualCount: 3; **Location:** country: Russia; stateProvince: Astrakhan'; locality: Dosang environs, fixed sands; decimalLatitude: 46.92; decimalLongitude: 47.92; **Event:** samplingProtocol: horse dung washing; eventDate: 2005-10-29; **Record Level:** collectionID: urn:lsid:biocol.org:col:34969; institutionCode: ZIN; collectionCode: Coleoptera
- **Type status:** Other material. **Occurrence:** recordedBy: V. Kozlov; individualCount: 6; **Location:** country: Russia; stateProvince: Astrakhan'; locality: 7 km NNE of Dosang; decimalLatitude: 46.93; decimalLongitude: 48.00; **Event:** samplingProtocol: horse dung washing; eventDate: 2005-10-31; **Record Level:** collectionID: urn:lsid:biocol.org:col:34969; institutionCode: ZIN; collectionCode: Coleoptera
- **Type status:** Other material. **Occurrence:** recordedBy: V. Kozlov; individualCount: 6; **Location:** country: Russia; stateProvince: Astrakhan'; locality: 7 km NNE of Dosang; decimalLatitude: 46.93; decimalLongitude: 48.00; **Event:** samplingProtocol: horse dung washing; eventDate: 2005-11-01; **Record Level:** collectionID: urn:lsid:biocol.org:col:34969; institutionCode: ZIN; collectionCode: Coleoptera
- **Type status:** Other material. **Occurrence:** recordedBy: A. V. Frolov, L. A. Akhmetova; individualCount: 3; **Location:** country: Russia; stateProvince: Astrakhan'; locality: Dosang environs, fixed sands; decimalLatitude: 46.92; decimalLongitude: 47.92; **Event:** samplingProtocol: old horse dung, larvae hand collecting; eventDate: 2008-04-09; **Record Level:** collectionID: urn:lsid:biocol.org:col:34969; institutionCode: ZIN; collectionCode: Coleoptera

##### Ecological interactions

###### Feeds on

Adults and larvae feed on horse dung.

##### Distribution

The species was recorded from Caucasus, Turkey, Iran, and Middle Asia (Nikolajev 1987). The range of it is unclear since it is difficult to separate from the closely-related and very similar *Aphodius melanostictus*. Some published records of *Aphodius clathratus* apparently belong to *Aphodius melanostictus*.

#### Aphodius coenosus

(Panzer, 1798)

##### Materials

- **Type status:** Other material. **Occurrence:** recordedBy: A. V. Frolov, L. A. Akhmetova; individualCount: 1; **Location:** country: Russia; stateProvince: Astrakhan'; locality: Dosang environs, fixed sands; decimalLatitude: 46.92; decimalLongitude: 47.92; **Event:** samplingProtocol: horse dung washing; eventDate: 2007-04-17; **Record Level:** collectionID: urn:lsid:biocol.org:col:34969; institutionCode: ZIN; collectionCode: Coleoptera
- **Type status:** Other material. **Occurrence:** recordedBy: A. V. Frolov, L. A. Akhmetova; individualCount: 1; **Location:** country: Russia; stateProvince: Astrakhan'; locality: 15 km NE of Dosang; decimalLatitude: 47.00; decimalLongitude: 47.98; **Event:** samplingProtocol: horse dung washing; eventDate: 2008-05-07; **Record Level:** collectionID: urn:lsid:biocol.org:col:34969; institutionCode: ZIN; collectionCode: Coleoptera
- **Type status:** Other material. **Occurrence:** recordedBy: A. V. Frolov, L. A. Akhmetova; individualCount: 1; **Location:** country: Russia; stateProvince: Astrakhan'; locality: Dosang environs, left bank of Akhtuba River, floodplain; decimalLatitude: 46.91; decimalLongitude: 47.91; **Event:** samplingProtocol: horse dung washing; eventDate: 2008-05-07; **Record Level:** collectionID: urn:lsid:biocol.org:col:34969; institutionCode: ZIN; collectionCode: Coleoptera

##### Ecological interactions

###### Feeds on

Cattle and wild herbivore dung.

##### Distribution

Europe (except for northernmost part), Asia Minor.

#### Aphodius curtulus

(Harold, 1866)

##### Materials

- **Type status:** Other material. **Occurrence:** recordedBy: A. V. Frolov, L. A. Akhmetova; individualCount: 2; **Location:** country: Russia; stateProvince: Astrakhan'; locality: 15 km NE of Dosang; decimalLatitude: 47.00; decimalLongitude: 47.98; **Event:** samplingProtocol: sand sifting; eventDate: 2008-04-07; **Record Level:** collectionID: urn:lsid:biocol.org:col:34969; institutionCode: ZIN; collectionCode: Coleoptera
- **Type status:** Other material. **Occurrence:** recordedBy: A. V. Frolov, L. A. Akhmetova; individualCount: 3; **Location:** country: Russia; stateProvince: Astrakhan'; locality: Dosang environs, fixed sands; decimalLatitude: 46.92; decimalLongitude: 47.92; **Event:** samplingProtocol: sand sifting; eventDate: 2006-10-7/11; **Record Level:** collectionID: urn:lsid:biocol.org:col:34969; institutionCode: ZIN; collectionCode: Coleoptera
- **Type status:** Other material. **Occurrence:** recordedBy: A. V. Frolov, L. A. Akhmetova; individualCount: 1; **Location:** country: Russia; stateProvince: Astrakhan'; locality: Dosang environs, fixed sands; decimalLatitude: 46.92; decimalLongitude: 47.92; **Event:** samplingProtocol: sand sifting; eventDate: 2007-04-13/15; **Record Level:** collectionID: urn:lsid:biocol.org:col:34969; institutionCode: ZIN; collectionCode: Coleoptera
- **Type status:** Other material. **Occurrence:** recordedBy: A. V. Frolov, L. A. Akhmetova; individualCount: 5; **Location:** country: Russia; stateProvince: Astrakhan'; locality: 8 km NE of Dosang; decimalLatitude: 46.94; decimalLongitude: 48.02; **Event:** samplingProtocol: sand sifting; eventDate: 2007-04-17/21; **Record Level:** collectionID: urn:lsid:biocol.org:col:34969; institutionCode: ZIN; collectionCode: Coleoptera

##### Ecological interactions

###### Feeds on

Adults were found in sand near plant roots.

##### Distribution

Caspian lowland desert.

#### Aphodius digitalis

D. Koshantschikov, 1894

##### Materials

- **Type status:** Other material. **Occurrence:** recordedBy: A. V. Frolov, L. A. Akhmetova; individualCount: 1; **Location:** country: Russia; stateProvince: Astrakhan'; locality: Dosang Railway Station; decimalLatitude: 46.90; decimalLongitude: 47.92; **Event:** samplingProtocol: light trap; eventDate: 2007-05-23; **Record Level:** collectionID: urn:lsid:biocol.org:col:34969; institutionCode: ZIN; collectionCode: Coleoptera

##### Distribution

Middle Asian deserts, Caspian lowland desert.

#### Aphodius distinctus

(Müller, 1776)

##### Materials

- **Type status:** Other material. **Occurrence:** recordedBy: A. V. Frolov, L. A. Akhmetova; individualCount: 1; **Location:** country: Russia; stateProvince: Astrakhan'; locality: Dosang environs, left bank of Akhtuba River, floodplain; decimalLatitude: 46.91; decimalLongitude: 47.91; **Event:** samplingProtocol: horse dung washing; eventDate: 2006-10-06; **Record Level:** collectionID: urn:lsid:biocol.org:col:34969; institutionCode: ZIN; collectionCode: Coleoptera
- **Type status:** Other material. **Occurrence:** recordedBy: A. V. Frolov, L. A. Akhmetova; individualCount: 15; **Location:** country: Russia; stateProvince: Astrakhan'; locality: Dosang environs, left bank of Akhtuba River, floodplain; decimalLatitude: 46.91; decimalLongitude: 47.91; **Event:** samplingProtocol: horse dung washing; eventDate: 2006-10-07; **Record Level:** collectionID: urn:lsid:biocol.org:col:34969; institutionCode: ZIN; collectionCode: Coleoptera
- **Type status:** Other material. **Occurrence:** recordedBy: A. V. Frolov, L. A. Akhmetova; individualCount: 9; **Location:** country: Russia; stateProvince: Astrakhan'; locality: Dosang environs, fixed sands; decimalLatitude: 46.92; decimalLongitude: 47.92; **Event:** samplingProtocol: horse dung washing; eventDate: 2006-10-09; **Record Level:** collectionID: urn:lsid:biocol.org:col:34969; institutionCode: ZIN; collectionCode: Coleoptera
- **Type status:** Other material. **Occurrence:** recordedBy: A. V. Frolov, L. A. Akhmetova; individualCount: 10; **Location:** country: Russia; stateProvince: Astrakhan'; locality: Dosang environs, fixed sands; decimalLatitude: 46.92; decimalLongitude: 47.92; **Event:** samplingProtocol: horse dung washing; eventDate: 2007-04-17; **Record Level:** collectionID: urn:lsid:biocol.org:col:34969; institutionCode: ZIN; collectionCode: Coleoptera
- **Type status:** Other material. **Occurrence:** recordedBy: A. V. Frolov; individualCount: 1; **Location:** country: Russia; stateProvince: Astrakhan'; locality: 15 km NE of Dosang; decimalLatitude: 47.00; decimalLongitude: 47.98; **Event:** samplingProtocol: cow dung washing; eventDate: 2010-10-12; **Record Level:** collectionID: urn:lsid:biocol.org:col:34969; institutionCode: ZIN; collectionCode: Coleoptera

##### Ecological interactions

###### Feeds on

Adults and larvae feed on cattle dung.

##### Distribution

The species is distributed throughout Europe, Transcaucasia, Central Asia, Northern Africa; it was introduced to North America.

#### Aphodius dosangi

Frolov et Akhmetova, 2008

##### Materials

- **Type status:** Holotype. **Occurrence:** recordedBy: A. V. Frolov, L. A. Akhmetova; individualCount: 1; sex: male; **Location:** country: Russia; stateProvince: Astrakhan'; locality: Dosang environs, fixed sands; decimalLatitude: 46.90; decimalLongitude: 47.92; **Event:** samplingProtocol: light trap; eventDate: 2007-05-24; **Record Level:** collectionID: urn:lsid:biocol.org:col:34969; institutionCode: ZIN; collectionCode: Coleoptera
- **Type status:** Paratype. **Occurrence:** recordedBy: A. V. Frolov, L. A. Akhmetova; individualCount: 5; sex: females; **Location:** country: Russia; stateProvince: Astrakhan'; locality: Dosang environs, fixed sands; decimalLatitude: 46.90; decimalLongitude: 47.92; **Event:** samplingProtocol: light trap; eventDate: 2007-05-24; **Record Level:** collectionID: urn:lsid:biocol.org:col:34969; institutionCode: ZIN; collectionCode: Coleoptera
- **Type status:** Paratype. **Occurrence:** recordedBy: A. V. Frolov, L. A. Akhmetova; individualCount: 2; sex: females; **Location:** country: Russia; stateProvince: Astrakhan'; locality: Dosang environs, left bank of Akhtuba River, floodplain; decimalLatitude: 46.91; decimalLongitude: 47.91; **Event:** samplingProtocol: cow dung washing; eventDate: 2006-10-06; **Record Level:** collectionID: urn:lsid:biocol.org:col:34969; institutionCode: ZIN; collectionCode: Coleoptera
- **Type status:** Paratype. **Occurrence:** recordedBy: A. V. Frolov, L. A. Akhmetova; individualCount: 2; sex: 1 male and 1 female; **Location:** country: Russia; stateProvince: Astrakhan'; locality: Dosang environs, fixed sands; decimalLatitude: 46.90; decimalLongitude: 47.92; **Event:** samplingProtocol: horse dung washing; eventDate: 2006-10-11; **Record Level:** collectionID: urn:lsid:biocol.org:col:34969; institutionCode: ZIN; collectionCode: Coleoptera
- **Type status:** Paratype. **Occurrence:** recordedBy: A. V. Frolov, L. A. Akhmetova; individualCount: 1; sex: female; **Location:** country: Russia; stateProvince: Astrakhan'; locality: Dosang environs, fixed sands; decimalLatitude: 46.92; decimalLongitude: 47.92; **Event:** samplingProtocol: hand collecting from old horse dung; eventDate: 2007-04-13/15; **Record Level:** collectionID: urn:lsid:biocol.org:col:34969; institutionCode: ZIN; collectionCode: Coleoptera
- **Type status:** Paratype. **Occurrence:** recordedBy: A. V. Frolov, L. A. Akhmetova; individualCount: 21; sex: 12 males and 9 females; **Location:** country: Russia; stateProvince: Astrakhan'; locality: Dosang environs, fixed sands; decimalLatitude: 46.92; decimalLongitude: 47.92; **Event:** samplingProtocol: larvae hand collecting old horse dung; eventDate: 2007-04-17; **Record Level:** collectionID: urn:lsid:biocol.org:col:34969; institutionCode: ZIN; collectionCode: Coleoptera
- **Type status:** Other material. **Occurrence:** recordedBy: A. V. Frolov, L. A. Akhmetova; individualCount: 2; **Location:** country: Russia; stateProvince: Astrakhan'; locality: Dosang environs, left bank of Akhtuba River, floodplain; decimalLatitude: 46.91; decimalLongitude: 47.91; **Event:** samplingProtocol: horse dung washing; eventDate: 2007-04-10; **Record Level:** collectionID: urn:lsid:biocol.org:col:34969; institutionCode: ZIN; collectionCode: Coleoptera
- **Type status:** Other material. **Occurrence:** recordedBy: A. V. Frolov, L. A. Akhmetova; individualCount: 3; **Location:** country: Russia; stateProvince: Astrakhan'; locality: Dosang environs, fixed sands; decimalLatitude: 46.92; decimalLongitude: 47.92; **Event:** samplingProtocol: horse dung washing; eventDate: 2007-04-17; **Record Level:** collectionID: urn:lsid:biocol.org:col:34969; institutionCode: ZIN; collectionCode: Coleoptera
- **Type status:** Other material. **Occurrence:** recordedBy: A. V. Frolov, L. A. Akhmetova; individualCount: 12; **Location:** country: Russia; stateProvince: Astrakhan'; locality: 8 km NE of Dosang; decimalLatitude: 46.94; decimalLongitude: 48.02; **Event:** samplingProtocol: larvae hand collecting old horse dung; eventDate: 2008-04-09; **Record Level:** collectionID: urn:lsid:biocol.org:col:34969; institutionCode: ZIN; collectionCode: Coleoptera
- **Type status:** Other material. **Occurrence:** recordedBy: A. V. Frolov, L. A. Akhmetova; individualCount: 3; **Location:** country: Russia; stateProvince: Astrakhan'; locality: Dosang environs, fixed sands; decimalLatitude: 46.92; decimalLongitude: 47.92; **Event:** samplingProtocol: cow dung washing; eventDate: 2012-05-15; **Record Level:** collectionID: urn:lsid:biocol.org:col:34969; institutionCode: ZIN; collectionCode: Coleoptera

##### Ecological interactions

###### Feeds on

Adults were found in horse and cow dung.

##### Distribution

Caspian lowland desert.

#### Aphodius erraticus

(Linnaeus, 1758)

##### Materials

- **Type status:** Other material. **Occurrence:** recordedBy: A. V. Frolov, L. A. Akhmetova; individualCount: 1; **Location:** country: Russia; stateProvince: Astrakhan'; locality: Dosang environs, left bank of Akhtuba River, floodplain; decimalLatitude: 46.91; decimalLongitude: 47.91; **Event:** samplingProtocol: horse dung washing; eventDate: 2012-05-13; **Record Level:** collectionID: urn:lsid:biocol.org:col:34969; institutionCode: ZIN; collectionCode: Coleoptera

##### Ecological interactions

###### Feeds on

Cattle and wild herbivore dung.

##### Distribution

Transpalearctic species, introduced to North America.

#### Aphodius fimetarius

(Linnaeus, 1758)

##### Materials

- **Type status:** Other material. **Occurrence:** recordedBy: A. V. Frolov, L. A. Akhmetova; individualCount: 2; **Location:** country: Russia; stateProvince: Astrakhan'; locality: Dosang environs, fixed sands; decimalLatitude: 46.92; decimalLongitude: 47.92; **Event:** samplingProtocol: horse dung washing; eventDate: 2006-10-09; **Record Level:** collectionID: urn:lsid:biocol.org:col:34969; institutionCode: ZIN; collectionCode: Coleoptera

##### Ecological interactions

###### Feeds on

Cattle and wild herbivore dung.

##### Distribution

Transpalearctic species, introduced in North America and Australia.

#### Aphodius frater

Mulsant et Rey, 1870

##### Materials

- **Type status:** Other material. **Occurrence:** recordedBy: A. V. Frolov, L. A. Akhmetova; individualCount: 3; **Location:** country: Russia; stateProvince: Astrakhan'; locality: Dosang environs, fixed sands; decimalLatitude: 46.92; decimalLongitude: 47.92; **Event:** samplingProtocol: hand collecting from old horse dung; eventDate: 2007-04-16; **Record Level:** collectionID: urn:lsid:biocol.org:col:34969; institutionCode: ZIN; collectionCode: Coleoptera
- **Type status:** Other material. **Occurrence:** recordedBy: A. V. Frolov, L. A. Akhmetova; individualCount: 15; **Location:** country: Russia; stateProvince: Astrakhan'; locality: Dosang environs, left bank of Akhtuba River, floodplain; decimalLatitude: 46.91; decimalLongitude: 47.91; **Event:** samplingProtocol: hand collecting from old horse dung; eventDate: 2008-04-03; **Record Level:** collectionID: urn:lsid:biocol.org:col:34969; institutionCode: ZIN; collectionCode: Coleoptera
- **Type status:** Other material. **Occurrence:** recordedBy: A. V. Frolov, L. A. Akhmetova; individualCount: 2; **Location:** country: Russia; stateProvince: Astrakhan'; locality: Dosang environs, fixed sands; decimalLatitude: 46.92; decimalLongitude: 47.92; **Event:** samplingProtocol: hand collecting from old horse dung; eventDate: 2007-04-13/15; **Record Level:** collectionID: urn:lsid:biocol.org:col:34969; institutionCode: ZIN; collectionCode: Coleoptera

##### Ecological interactions

###### Feeds on

Adults feed on dry cattle dung.

##### Distribution

Western Palearctic region South Europe the south-west to West Siberia in the north-east.

#### Aphodius granarius

(Linnaeus, 1767)

##### Materials

- **Type status:** Other material. **Occurrence:** recordedBy: A. V. Frolov, L. A. Akhmetova; individualCount: 6; **Location:** country: Russia; stateProvince: Astrakhan'; locality: Dosang environs, left bank of Akhtuba River, floodplain; decimalLatitude: 46.91; decimalLongitude: 47.91; **Event:** samplingProtocol: horse dung washing; eventDate: 2007-04-10; **Record Level:** collectionID: urn:lsid:biocol.org:col:34969; institutionCode: ZIN; collectionCode: Coleoptera
- **Type status:** Other material. **Occurrence:** recordedBy: A. V. Frolov, L. A. Akhmetova; individualCount: 6; **Location:** country: Russia; stateProvince: Astrakhan'; locality: Dosang environs, right bank of Akhtuba River, floodplain; decimalLatitude: 46.90; decimalLongitude: 47.90; **Event:** samplingProtocol: horse dung washing; eventDate: 2007-05-12; **Record Level:** collectionID: urn:lsid:biocol.org:col:34969; institutionCode: ZIN; collectionCode: Coleoptera
- **Type status:** Other material. **Occurrence:** recordedBy: A. V. Frolov, L. A. Akhmetova; individualCount: 1; **Location:** country: Russia; stateProvince: Astrakhan'; locality: Dosang environs, left bank of Akhtuba River, floodplain; decimalLatitude: 46.91; decimalLongitude: 47.91; **Event:** samplingProtocol: horse dung washing; eventDate: 2008-05-05; **Record Level:** collectionID: urn:lsid:biocol.org:col:34969; institutionCode: ZIN; collectionCode: Coleoptera
- **Type status:** Other material. **Occurrence:** recordedBy: A. V. Frolov, L. A. Akhmetova; individualCount: 2; **Location:** country: Russia; stateProvince: Astrakhan'; locality: 15 km NE of Dosang; decimalLatitude: 47.00; decimalLongitude: 47.98; **Event:** samplingProtocol: horse dung washing; eventDate: 2008-05-07; **Record Level:** collectionID: urn:lsid:biocol.org:col:34969; institutionCode: ZIN; collectionCode: Coleoptera

##### Ecological interactions

###### Feeds on

Coprophagous generalist.

##### Distribution

West Palearctic region (except for northernmost part), introduced in North America.

#### Aphodius gregarius

Harold, 1871

##### Materials

- **Type status:** Other material. **Occurrence:** recordedBy: A. V. Frolov, L. A. Akhmetova; individualCount: 1; **Taxon:** scientificName: gregarius; **Location:** country: Russia; stateProvince: Astrakhan'; locality: Dosang Railway Station; decimalLatitude: 46.90; decimalLongitude: 47.92; **Event:** samplingProtocol: light trap; eventDate: 2007-05-23; **Record Level:** collectionID: urn:lsid:biocol.org:col:34969; institutionCode: ZIN; collectionCode: Coleoptera

##### Ecological interactions

###### Feeds on

Cattle dung.

##### Distribution

Steppe and semidesert zones from Lower Volga in the west to Mongolia in the east.

#### Aphodius gresseri

Semenov, 1898

##### Materials

- **Type status:** Other material. **Occurrence:** recordedBy: A. V. Frolov, L. A. Akhmetova; individualCount: 2; **Location:** country: Russia; stateProvince: Astrakhan'; locality: Dosang environs, fixed sands; decimalLatitude: 46.91; decimalLongitude: 47.91; **Event:** samplingProtocol: horse dung washing; eventDate: 2007-04-10; **Record Level:** collectionID: urn:lsid:biocol.org:col:34969; institutionCode: ZIN; collectionCode: Coleoptera
- **Type status:** Other material. **Occurrence:** recordedBy: K. V. Makarov; individualCount: 1; **Location:** country: Russia; stateProvince: Astrakhan'; locality: 1.5-5 km NE of Dosang; **Event:** samplingProtocol: horse dung washing; eventDate: 2007-04-14; **Record Level:** collectionID: urn:lsid:biocol.org:col:34969; institutionCode: ZIN; collectionCode: Coleoptera
- **Type status:** Other material. **Occurrence:** recordedBy: A. V. Frolov, L. A. Akhmetova; individualCount: 2; **Location:** country: Russia; stateProvince: Astrakhan'; locality: Dosang environs, fixed sands; decimalLatitude: 46.92; decimalLongitude: 47.92; **Event:** samplingProtocol: larvae hand collecting old horse dung; eventDate: 2007-04-16; **Record Level:** collectionID: urn:lsid:biocol.org:col:34969; institutionCode: ZIN; collectionCode: Coleoptera
- **Type status:** Other material. **Occurrence:** recordedBy: A. V. Frolov, L. A. Akhmetova; individualCount: 2; **Location:** country: Russia; stateProvince: Astrakhan'; locality: Dosang environs, fixed sands; decimalLatitude: 46.92; decimalLongitude: 47.92; **Event:** samplingProtocol: horse dung washing; eventDate: 2007-04-17; **Record Level:** collectionID: urn:lsid:biocol.org:col:34969; institutionCode: ZIN; collectionCode: Coleoptera
- **Type status:** Other material. **Occurrence:** recordedBy: A. V. Frolov, L. A. Akhmetova; individualCount: 10; **Location:** country: Russia; stateProvince: Astrakhan'; locality: Dosang environs, fixed sands; decimalLatitude: 46.92; decimalLongitude: 47.92; **Event:** samplingProtocol: larvae hand collecting old horse dung; eventDate: 2007-04-17; **Record Level:** collectionID: urn:lsid:biocol.org:col:34969; institutionCode: ZIN; collectionCode: Coleoptera
- **Type status:** Other material. **Occurrence:** recordedBy: A. V. Frolov, L. A. Akhmetova; individualCount: 2; **Location:** country: Russia; stateProvince: Astrakhan'; locality: Dosang environs, fixed sands; decimalLatitude: 46.92; decimalLongitude: 47.92; **Event:** samplingProtocol: larvae hand collecting old horse dung; eventDate: 2007-04-21; **Record Level:** collectionID: urn:lsid:biocol.org:col:34969; institutionCode: ZIN; collectionCode: Coleoptera

##### Ecological interactions

###### Feeds on

Adults and larvae feed on cattle dung.

##### Distribution

Volga Region up to Vladimir Province in the north. Kazakh steppe up to Sultankeldy Lake in the East.

#### Aphodius hauseri

(Reitter, 1894)

##### Materials

- **Type status:** Other material. **Occurrence:** recordedBy: A. V. Frolov, L. A. Akhmetova; individualCount: 5; **Location:** country: Russia; stateProvince: Astrakhan'; locality: 2 km NE of Dosang; decimalLatitude: 46.92; decimalLongitude: 47.93; **Event:** samplingProtocol: larvae hand collecting from sand; eventDate: 2007-05-01; **Record Level:** collectionID: urn:lsid:biocol.org:col:34969; institutionCode: ZIN; collectionCode: Coleoptera
- **Type status:** Other material. **Occurrence:** recordedBy: A. V. Frolov, L. A. Akhmetova; individualCount: 2; **Location:** country: Russia; stateProvince: Astrakhan'; locality: 15 km NE of Dosang; decimalLatitude: 47.00; decimalLongitude: 47.98; **Event:** samplingProtocol: sand sifting; eventDate: 2008-05-07; **Record Level:** collectionID: urn:lsid:biocol.org:col:34969; institutionCode: ZIN; collectionCode: Coleoptera

##### Distribution

Middle Asian deserts.

#### Aphodius hydrochaeris

(Fabricius, 1798)

##### Materials

- **Type status:** Other material. **Occurrence:** recordedBy: A. V. Frolov, L. A. Akhmetova; individualCount: 3; **Location:** country: Russia; stateProvince: Astrakhan'; locality: Dosang environs, left bank of Akhtuba River, floodplain; decimalLatitude: 46.91; decimalLongitude: 47.91; **Event:** samplingProtocol: cow dung washing; eventDate: 2006-10-06; **Record Level:** collectionID: urn:lsid:biocol.org:col:34969; institutionCode: ZIN; collectionCode: Coleoptera
- **Type status:** Other material. **Occurrence:** recordedBy: A. V. Frolov, L. A. Akhmetova; individualCount: 1; **Location:** country: Russia; stateProvince: Astrakhan'; locality: Dosang Railway Station; decimalLatitude: 46.90; decimalLongitude: 47.92; **Event:** samplingProtocol: light trap; eventDate: 2007-05-23; **Record Level:** collectionID: urn:lsid:biocol.org:col:34969; institutionCode: ZIN; collectionCode: Coleoptera
- **Type status:** Other material. **Occurrence:** recordedBy: A. V. Frolov, L. A. Akhmetova; individualCount: 1; **Location:** country: Russia; stateProvince: Astrakhan'; locality: Dosang environs, left bank of Akhtuba River, floodplain; decimalLatitude: 46.91; decimalLongitude: 47.91; **Event:** samplingProtocol: horse dung washing; eventDate: 2012-05-13; **Record Level:** collectionID: urn:lsid:biocol.org:col:34969; institutionCode: ZIN; collectionCode: Coleoptera

##### Ecological interactions

###### Feeds on

Horse dung.

##### Distribution

West Palearctic region (except for northernmost part).

#### Aphodius ictericus

(Laicharting, 1781)

##### Materials

- **Type status:** Other material. **Occurrence:** recordedBy: A. V. Frolov, L. A. Akhmetova; individualCount: 4; **Location:** country: Russia; stateProvince: Astrakhan'; locality: Dosang environs, left bank of Akhtuba River, floodplain; decimalLatitude: 46.91; decimalLongitude: 47.91; **Event:** samplingProtocol: cow dung washing; eventDate: 2006-10-06; **Record Level:** collectionID: urn:lsid:biocol.org:col:34969; institutionCode: ZIN; collectionCode: Coleoptera
- **Type status:** Other material. **Occurrence:** recordedBy: A. V. Frolov, L. A. Akhmetova; individualCount: 10; **Location:** country: Russia; stateProvince: Astrakhan'; locality: Dosang environs, left bank of Akhtuba River, floodplain; decimalLatitude: 46.91; decimalLongitude: 47.91; **Event:** samplingProtocol: cow dung washing; eventDate: 2006-10-07; **Record Level:** collectionID: urn:lsid:biocol.org:col:34969; institutionCode: ZIN; collectionCode: Coleoptera
- **Type status:** Other material. **Occurrence:** recordedBy: A. V. Frolov, L. A. Akhmetova; individualCount: 34; **Location:** country: Russia; stateProvince: Astrakhan'; locality: Dosang environs, fixed sands; decimalLatitude: 46.92; decimalLongitude: 47.92; **Event:** samplingProtocol: cow dung washing; eventDate: 2012-05-15; **Record Level:** collectionID: urn:lsid:biocol.org:col:34969; institutionCode: ZIN; collectionCode: Coleoptera

##### Ecological interactions

###### Feeds on

Cattle dung.

##### Distribution

Distributed throughout Europe (except for northernmost part), in North Africa, Asia Minor, Iran, up to Irtysh River in the east.

#### Aphodius immundus

Creutzer, 1799

##### Materials

- **Type status:** Other material. **Occurrence:** recordedBy: A. V. Frolov, L. A. Akhmetova; individualCount: 10; **Location:** country: Russia; stateProvince: Astrakhan'; locality: Dosang environs, left bank of Akhtuba River, floodplain; decimalLatitude: 46.91; decimalLongitude: 47.91; **Event:** samplingProtocol: cow dung washing; eventDate: 2006-10-06; **Record Level:** collectionID: urn:lsid:biocol.org:col:34969; institutionCode: ZIN; collectionCode: Coleoptera
- **Type status:** Other material. **Occurrence:** recordedBy: A. V. Frolov, L. A. Akhmetova; individualCount: 10; **Location:** country: Russia; stateProvince: Astrakhan'; locality: Dosang environs, fixed sands; decimalLatitude: 46.92; decimalLongitude: 47.92; **Event:** samplingProtocol: cow dung washing; eventDate: 2007-04-16; **Record Level:** collectionID: urn:lsid:biocol.org:col:34969; institutionCode: ZIN; collectionCode: Coleoptera
- **Type status:** Other material. **Occurrence:** recordedBy: A. V. Frolov, L. A. Akhmetova; individualCount: 4; **Location:** country: Russia; stateProvince: Astrakhan'; locality: Dosang Railway Station; decimalLatitude: 46.90; decimalLongitude: 47.92; **Event:** samplingProtocol: light trap; eventDate: 2007-05-23; **Record Level:** collectionID: urn:lsid:biocol.org:col:34969; institutionCode: ZIN; collectionCode: Coleoptera
- **Type status:** Other material. **Occurrence:** recordedBy: A. V. Frolov, L. A. Akhmetova; individualCount: 1; **Location:** country: Russia; stateProvince: Astrakhan'; locality: Dosang environs, left bank of Akhtuba River, floodplain; decimalLatitude: 46.91; decimalLongitude: 47.91; **Event:** samplingProtocol: horse dung washing; eventDate: 2012-05-13; **Record Level:** collectionID: urn:lsid:biocol.org:col:34969; institutionCode: ZIN; collectionCode: Coleoptera
- **Type status:** Other material. **Occurrence:** recordedBy: A. V. Frolov, L. A. Akhmetova; individualCount: 2; **Location:** country: Russia; stateProvince: Astrakhan'; locality: Dosang environs, fixed sands; decimalLatitude: 46.92; decimalLongitude: 47.92; **Event:** samplingProtocol: cow dung washing; eventDate: 2012-05-15; **Record Level:** collectionID: urn:lsid:biocol.org:col:34969; institutionCode: ZIN; collectionCode: Coleoptera

##### Ecological interactions

###### Feeds on

Cattle dung

##### Distribution

Western Palearctic region from North Africa in the south-west to Baikal Lake in the north-east.

#### Aphodius kizeritskyi

Frolov, 2002

##### Materials

- **Type status:** Other material. **Occurrence:** recordedBy: V. Kozlov; individualCount: 22; **Location:** country: Russia; stateProvince: Astrakhan'; locality: 7 km NNE of Dosang; **Event:** samplingProtocol: horse dung washing; eventDate: 2005-10-31; **Record Level:** collectionID: urn:lsid:biocol.org:col:34969; institutionCode: ZIN; collectionCode: Coleoptera
- **Type status:** Other material. **Occurrence:** recordedBy: V. Kozlov; individualCount: 35; **Location:** country: Russia; stateProvince: Astrakhan'; locality: 7 km NNE of Dosang; decimalLatitude: 46.93; decimalLongitude: 48.00; **Event:** samplingProtocol: horse dung washing; eventDate: 2005-11-01; **Record Level:** collectionID: urn:lsid:biocol.org:col:34969; institutionCode: ZIN; collectionCode: Coleoptera
- **Type status:** Other material. **Occurrence:** recordedBy: A. V. Frolov; individualCount: 36; **Location:** country: Russia; stateProvince: Astrakhan'; locality: Dosang environs, fixed sands; decimalLatitude: 46.92; decimalLongitude: 47.92; **Event:** samplingProtocol: horse dung washing; eventDate: 2005-11-01; **Record Level:** collectionID: urn:lsid:biocol.org:col:34969; institutionCode: ZIN; collectionCode: Coleoptera
- **Type status:** Other material. **Occurrence:** recordedBy: A. V. Frolov; individualCount: 48; **Location:** country: Russia; stateProvince: Astrakhan'; locality: 15 km NE of Dosang; decimalLatitude: 47.00; decimalLongitude: 47.98; **Event:** samplingProtocol: horse dung washing; eventDate: 2009-10-31; **Record Level:** collectionID: urn:lsid:biocol.org:col:34969; institutionCode: ZIN; collectionCode: Coleoptera
- **Type status:** Other material. **Occurrence:** recordedBy: A. V. Frolov; individualCount: 9; **Location:** country: Russia; stateProvince: Astrakhan'; locality: 8 km NE of Dosang; decimalLatitude: 46.94; decimalLongitude: 48.02; **Event:** samplingProtocol: horse dung washing; eventDate: 2009-11-04; **Record Level:** collectionID: urn:lsid:biocol.org:col:34969; institutionCode: ZIN; collectionCode: Coleoptera

##### Ecological interactions

###### Feeds on

Horse dung.

##### Distribution

Caspian lowland desert, Middle Asian deserts.

#### Aphodius kisilkumi

Solsky, 1876

##### Materials

- **Type status:** Other material. **Occurrence:** recordedBy: A. V. Frolov, L. A. Akhmetova; individualCount: 16; **Location:** country: Russia; stateProvince: Astrakhan'; locality: Dosang environs, fixed sands; decimalLatitude: 46.92; decimalLongitude: 47.92; **Event:** samplingProtocol: cow dung washing; eventDate: 2012-05-15; **Record Level:** collectionID: urn:lsid:biocol.org:col:34969; institutionCode: ZIN; collectionCode: Coleoptera

##### Ecological interactions

###### Feeds on

Cattle dung.

##### Distribution

Caspian lowland desert, Middle Asian deserts.

#### Aphodius kraatzi

Harold, 1868

##### Materials

- **Type status:** Other material. **Occurrence:** recordedBy: A. V. Frolov, L. A. Akhmetova; individualCount: 1; **Location:** country: Russia; stateProvince: Astrakhan'; locality: Dosang Railway Station; decimalLatitude: 46.90; decimalLongitude: 47.92; **Event:** samplingProtocol: light trap; eventDate: 2007-05-23; **Record Level:** collectionID: urn:lsid:biocol.org:col:34969; institutionCode: ZIN; collectionCode: Coleoptera
- **Type status:** Other material. **Occurrence:** recordedBy: A. V. Frolov, L. A. Akhmetova; individualCount: 16; **Location:** country: Russia; stateProvince: Astrakhan'; locality: Dosang Railway Station; decimalLatitude: 46.90; decimalLongitude: 47.92; **Event:** samplingProtocol: light trap; eventDate: 2007-05-23; **Record Level:** collectionID: urn:lsid:biocol.org:col:34969; institutionCode: ZIN; collectionCode: Coleoptera

##### Ecological interactions

###### Feeds on

Apparently saprophagous species.

##### Distribution

Distributed mostly in steppe and desert zones in South Europe, Caucasus, Asia Minor, Middle Asia.

#### Aphodius lividus

(Olivier, 1789)

##### Materials

- **Type status:** Other material. **Occurrence:** recordedBy: A. V. Frolov, L. A. Akhmetova; individualCount: 12; **Location:** country: Russia; stateProvince: Astrakhan'; locality: Dosang Railway Station; decimalLatitude: 46.90; decimalLongitude: 47.92; **Event:** samplingProtocol: light trap; eventDate: 2007-05-23; **Record Level:** collectionID: urn:lsid:biocol.org:col:34969; institutionCode: ZIN; collectionCode: Coleoptera
- **Type status:** Other material. **Occurrence:** recordedBy: A. V. Frolov, L. A. Akhmetova; individualCount: 1; **Location:** country: Russia; stateProvince: Astrakhan'; locality: 15 km NE of Dosang; decimalLatitude: 47.00; decimalLongitude: 47.98; **Event:** samplingProtocol: horse dung washing; eventDate: 2008-05-07; **Record Level:** collectionID: urn:lsid:biocol.org:col:34969; institutionCode: ZIN; collectionCode: Coleoptera
- **Type status:** Other material. **Occurrence:** recordedBy: A. V. Frolov, L. A. Akhmetova; individualCount: 15; **Location:** country: Russia; stateProvince: Astrakhan'; locality: Dosang environs, fixed sands; decimalLatitude: 46.92; decimalLongitude: 47.92; **Event:** samplingProtocol: cow dung washing; eventDate: 2012-05-15; **Record Level:** collectionID: urn:lsid:biocol.org:col:34969; institutionCode: ZIN; collectionCode: Coleoptera

##### Ecological interactions

###### Feeds on

Cattle dung.

##### Distribution

West Palearctic region (except for northernmost part).

#### Aphodius lugens

Creutzer, 1799

##### Materials

- **Type status:** Other material. **Occurrence:** recordedBy: A. V. Frolov, L. A. Akhmetova; individualCount: 3; **Location:** country: Russia; stateProvince: Astrakhan'; locality: Dosang environs, left bank of Akhtuba River, floodplain; decimalLatitude: 46.91; decimalLongitude: 47.91; **Event:** samplingProtocol: cow dung washing; eventDate: 2006-10-06; **Record Level:** collectionID: urn:lsid:biocol.org:col:34969; institutionCode: ZIN; collectionCode: Coleoptera
- **Type status:** Other material. **Occurrence:** recordedBy: A. V. Frolov, L. A. Akhmetova; individualCount: 1; **Location:** country: Russia; stateProvince: Astrakhan'; locality: Dosang environs, fixed sands; decimalLatitude: 46.92; decimalLongitude: 47.92; **Event:** samplingProtocol: cow dung washing; eventDate: 2012-05-15; **Record Level:** collectionID: urn:lsid:biocol.org:col:34969; institutionCode: ZIN; collectionCode: Coleoptera

##### Ecological interactions

###### Feeds on

Cattle dung.

##### Distribution

Western Palearctic region from North Africa in the south-west to Saur-Tarbagataj in the north-east.

#### Aphodius melanostictus

W. Schmidt, 1840

##### Materials

- **Type status:** Other material. **Occurrence:** recordedBy: V. Kozlov; individualCount: 25; **Location:** country: Russia; stateProvince: Astrakhan'; locality: 7 km NNE of Dosang; decimalLatitude: 46.93; decimalLongitude: 48.00; **Event:** samplingProtocol: horse dung washing; eventDate: 2005-11-01; **Record Level:** collectionID: urn:lsid:biocol.org:col:34969; institutionCode: ZIN; collectionCode: Coleoptera
- **Type status:** Other material. **Occurrence:** recordedBy: A. V. Frolov, L. A. Akhmetova; individualCount: 4; **Location:** country: Russia; stateProvince: Astrakhan'; locality: Dosang environs, fixed sands; decimalLatitude: 46.92; decimalLongitude: 47.92; **Event:** samplingProtocol: horse dung washing; eventDate: 2006-10-09; **Record Level:** collectionID: urn:lsid:biocol.org:col:34969; institutionCode: ZIN; collectionCode: Coleoptera
- **Type status:** Other material. **Occurrence:** recordedBy: A. V. Frolov, L. A. Akhmetova; individualCount: 15; **Location:** country: Russia; stateProvince: Astrakhan'; locality: Dosang environs, fixed sands; decimalLatitude: 46.92; decimalLongitude: 47.92; **Event:** samplingProtocol: horse dung washing; eventDate: 2007-04-17; **Record Level:** collectionID: urn:lsid:biocol.org:col:34969; institutionCode: ZIN; collectionCode: Coleoptera

##### Ecological interactions

###### Feeds on

Cattle dung.

##### Distribution

Western Palearctic region up to South Siberia in the north-east.

#### Aphodius multiplex

Reitter, 1897

##### Materials

- **Type status:** Other material. **Occurrence:** recordedBy: A. V. Frolov, L. A. Akhmetova; individualCount: 9; **Location:** country: Russia; stateProvince: Astrakhan'; locality: Dosang environs, fixed sands; decimalLatitude: 46.92; decimalLongitude: 47.92; **Event:** samplingProtocol: horse dung washing; eventDate: 2007-04-17; **Record Level:** collectionID: urn:lsid:biocol.org:col:34969; institutionCode: ZIN; collectionCode: Coleoptera
- **Type status:** Other material. **Occurrence:** recordedBy: A. V. Frolov, L. A. Akhmetova; individualCount: 11; **Location:** country: Russia; stateProvince: Astrakhan'; locality: Dosang environs, fixed sands; decimalLatitude: 46.92; decimalLongitude: 47.92; **Event:** samplingProtocol: horse dung washing; eventDate: 2012-05-15; **Record Level:** collectionID: urn:lsid:biocol.org:col:34969; institutionCode: ZIN; collectionCode: Coleoptera
- **Type status:** Other material. **Occurrence:** recordedBy: A. V. Frolov, L. A. Akhmetova; individualCount: 6; **Location:** country: Russia; stateProvince: Astrakhan'; locality: Dosang environs, fixed sands; decimalLatitude: 46.92; decimalLongitude: 47.92; **Event:** samplingProtocol: horse dung washing; eventDate: 2007-04-13/15; **Record Level:** collectionID: urn:lsid:biocol.org:col:34969; institutionCode: ZIN; collectionCode: Coleoptera

##### Ecological interactions

###### Feeds on

Cattle dung.

##### Distribution

Steppe and semidesert zones from Ciscaucasia in the west to Mongolia in the east.

#### Aphodius plustschewskii

D. Koshantschikov, 1894

##### Materials

- **Type status:** Other material. **Occurrence:** recordedBy: V. Kozlov; individualCount: 2; **Location:** country: Russia; stateProvince: Astrakhan'; locality: 7 km NNE of Dosang; **Event:** samplingProtocol: manually collected from horse dung; eventDate: 2005-10-31; **Record Level:** collectionID: urn:lsid:biocol.org:col:34969; institutionCode: ZIN; collectionCode: Coleoptera
- **Type status:** Other material. **Occurrence:** recordedBy: A. V. Frolov; individualCount: 37; **Location:** country: Russia; stateProvince: Astrakhan'; locality: Dosang environs, fixed sands; decimalLatitude: 46.92; decimalLongitude: 47.92; **Event:** samplingProtocol: manually collected from horse dung; eventDate: 2005-11-01; **Record Level:** collectionID: urn:lsid:biocol.org:col:34969; institutionCode: ZIN; collectionCode: Coleoptera
- **Type status:** Other material. **Occurrence:** recordedBy: V. Kozlov; individualCount: 2; **Location:** country: Russia; stateProvince: Astrakhan'; locality: 6 km NNE of Dosang; **Event:** samplingProtocol: manually collected from horse dung; eventDate: 2005-11-02; **Record Level:** collectionID: urn:lsid:biocol.org:col:34969; institutionCode: ZIN; collectionCode: Coleoptera
- **Type status:** Other material. **Occurrence:** recordedBy: V. Kozlov; individualCount: 2; **Location:** country: Russia; stateProvince: Astrakhan'; locality: 7 km NNE of Dosang; **Event:** samplingProtocol: manually collected from horse dung; eventDate: 2005-11-03; **Record Level:** collectionID: urn:lsid:biocol.org:col:34969; institutionCode: ZIN; collectionCode: Coleoptera
- **Type status:** Other material. **Occurrence:** recordedBy: A. V. Frolov; individualCount: 1; **Location:** country: Russia; stateProvince: Astrakhan'; locality: 15 km NE of Dosang; decimalLatitude: 47.00; decimalLongitude: 47.98; **Event:** samplingProtocol: manually collected from horse dung; eventDate: 2009-10-31; **Record Level:** collectionID: urn:lsid:biocol.org:col:34969; institutionCode: ZIN; collectionCode: Coleoptera
- **Type status:** Other material. **Occurrence:** recordedBy: A. V. Frolov; individualCount: 88; **Location:** country: Russia; stateProvince: Astrakhan'; locality: Dosang environs, fixed sands; **Event:** samplingProtocol: manually collected from horse dung; eventDate: 2009-11-02; **Record Level:** collectionID: urn:lsid:biocol.org:col:34969; institutionCode: ZIN; collectionCode: Coleoptera

##### Ecological interactions

###### Feeds on

**Figure 6.**
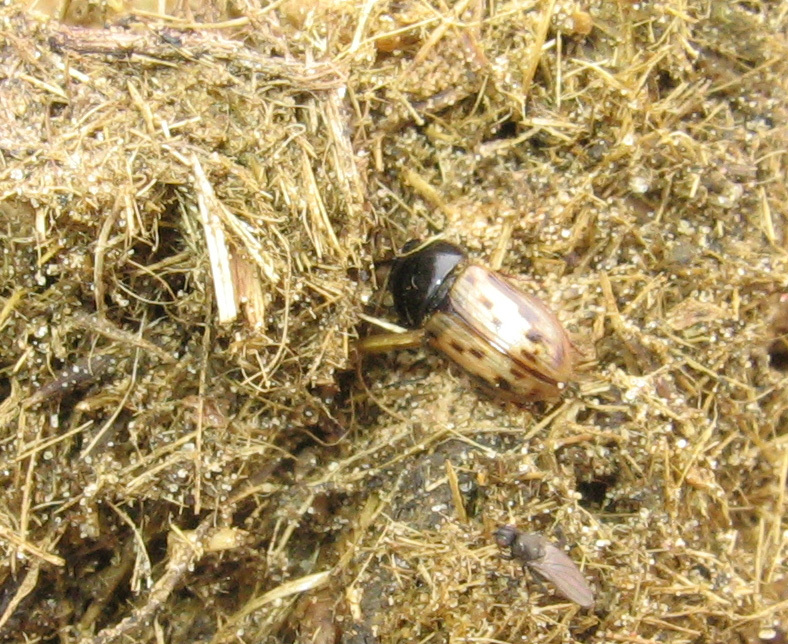
*Aphodius plustschewskii* in horse dung. Dosang environs, Astrakhan Province, Russia.

Horse dung (Fig. 6).

##### Distribution

Caspian lowland desert, Middle Asian deserts

#### Aphodius punctipennis

Erichson, 1848

##### Materials

- **Type status:** Other material. **Occurrence:** recordedBy: A. V. Frolov, L. A. Akhmetova; individualCount: 1; **Location:** country: Russia; stateProvince: Astrakhan'; locality: Dosang Railway Station; decimalLatitude: 46.90; decimalLongitude: 47.92; **Event:** samplingProtocol: light trap; eventDate: 2007-05-23; **Record Level:** collectionID: urn:lsid:biocol.org:col:34969; institutionCode: ZIN; collectionCode: Coleoptera

##### Ecological interactions

###### Feeds on

Cattle dung

##### Distribution

Southern Central Europe, Transcaucasus, Caspian lowland and Central Asian Deserts up to Dzhungar Alatau in the east

#### Aphodius quadriguttatus

(Herbst, 1783)

##### Materials

- **Type status:** Other material. **Occurrence:** recordedBy: A. V. Frolov, L. A. Akhmetova; individualCount: 6; **Location:** country: Russia; stateProvince: Astrakhan'; locality: Dosang environs, fixed sands; decimalLatitude: 46.92; decimalLongitude: 47.92; **Event:** samplingProtocol: cow dung washing; eventDate: 2007-04-17; **Record Level:** collectionID: urn:lsid:biocol.org:col:34969; institutionCode: ZIN; collectionCode: Coleoptera
- **Type status:** Other material. **Occurrence:** recordedBy: A. V. Frolov, L. A. Akhmetova; individualCount: 12; **Location:** country: Russia; stateProvince: Astrakhan'; locality: Dosang environs, fixed sands; decimalLatitude: 46.92; decimalLongitude: 47.92; **Event:** samplingProtocol: cow dung washing; eventDate: 2012-05-15; **Record Level:** collectionID: urn:lsid:biocol.org:col:34969; institutionCode: ZIN; collectionCode: Coleoptera

##### Ecological interactions

###### Feeds on

Cattle and wild herbivore dung.

##### Distribution

Western Palearctic region from North Africa in the south-west to Altai Mountains the north-east.

#### Aphodius rufipes

(Linnaeus, 1758)

##### Materials

- **Type status:** Other material. **Occurrence:** recordedBy: A. V. Frolov, L. A. Akhmetova; individualCount: 1; **Location:** country: Russia; stateProvince: Astrakhan'; locality: Dosang environs, left bank of Akhtuba River, floodplain; decimalLatitude: 46.91; decimalLongitude: 47.91; **Event:** samplingProtocol: cow dung washing; eventDate: 2006-10-06; **Record Level:** collectionID: urn:lsid:biocol.org:col:34969; institutionCode: ZIN; collectionCode: Coleoptera

##### Ecological interactions

###### Feeds on

Cattle and wild herbivore dung.

##### Distribution

Transpalearctic species.

#### Aphodius satellitius

(Herbst, 1789)

##### Materials

- **Type status:** Other material. **Occurrence:** recordedBy: A. V. Frolov, L. A. Akhmetova; individualCount: 2; **Location:** country: Russia; stateProvince: Astrakhan'; locality: Dosang environs, fixed sands; decimalLatitude: 46.92; decimalLongitude: 47.92; **Event:** samplingProtocol: horse dung washing; eventDate: 2007-04-17; **Record Level:** collectionID: urn:lsid:biocol.org:col:34969; institutionCode: ZIN; collectionCode: Coleoptera
- **Type status:** Other material. **Occurrence:** recordedBy: A. V. Frolov, L. A. Akhmetova; individualCount: 14; **Location:** country: Russia; stateProvince: Astrakhan'; locality: Dosang environs, right bank of Akhtuba River, floodplain; decimalLatitude: 46.90; decimalLongitude: 47.90; **Event:** samplingProtocol: horse dung washing; eventDate: 2007-05-23; **Record Level:** collectionID: urn:lsid:biocol.org:col:34969; institutionCode: ZIN; collectionCode: Coleoptera
- **Type status:** Other material. **Occurrence:** recordedBy: A. V. Frolov, L. A. Akhmetova; individualCount: 1; **Location:** country: Russia; stateProvince: Astrakhan'; locality: Dosang environs, left bank of Akhtuba River, floodplain; decimalLatitude: 46.91; decimalLongitude: 47.91; **Event:** samplingProtocol: horse dung washing; eventDate: 2012-05-13; **Record Level:** collectionID: urn:lsid:biocol.org:col:34969; institutionCode: ZIN; collectionCode: Coleoptera
- **Type status:** Other material. **Occurrence:** recordedBy: A. V. Frolov, L. A. Akhmetova; individualCount: 1; **Location:** country: Russia; stateProvince: Astrakhan'; locality: Dosang environs, fixed sands; decimalLatitude: 46.92; decimalLongitude: 47.92; **Event:** samplingProtocol: horse dung washing; eventDate: 2007-04-13/15; **Record Level:** collectionID: urn:lsid:biocol.org:col:34969; institutionCode: ZIN; collectionCode: Coleoptera

##### Ecological interactions

###### Feeds on

Cattle dung.

##### Distribution

Western Palearctic region from North Africa in the south-west to southern Urals in the north-east. Mostly in steppe zones.

#### Aphodius scrofa

(Fabricius, 1787)

##### Materials

- **Type status:** Other material. **Occurrence:** recordedBy: A. V. Frolov, L. A. Akhmetova; individualCount: 4; **Location:** country: Russia; stateProvince: Astrakhan'; locality: Dosang environs, left bank of Akhtuba River, floodplain; decimalLatitude: 46.91; decimalLongitude: 47.91; **Event:** samplingProtocol: horse dung washing; eventDate: 2007-04-10; **Record Level:** collectionID: urn:lsid:biocol.org:col:34969; institutionCode: ZIN; collectionCode: Coleoptera

##### Ecological interactions

###### Feeds on

Cattle and wild herbivore dung.

##### Distribution

Transpalearctic species occurring mostly in steppe zone.

#### Aphodius serotinus

(Panzer, 1799)

##### Materials

- **Type status:** Other material. **Occurrence:** recordedBy: A. V. Frolov, L. A. Akhmetova; individualCount: 3; **Location:** country: Russia; stateProvince: Astrakhan'; locality: Dosang environs, left bank of Akhtuba River, floodplain; decimalLatitude: 46.91; decimalLongitude: 47.91; **Event:** samplingProtocol: cow dung washing; eventDate: 2006-10-06; **Record Level:** collectionID: urn:lsid:biocol.org:col:34969; institutionCode: ZIN; collectionCode: Coleoptera
- **Type status:** Other material. **Occurrence:** recordedBy: A. V. Frolov, L. A. Akhmetova; individualCount: 18; **Location:** country: Russia; stateProvince: Astrakhan'; locality: Dosang environs, left bank of Akhtuba River, floodplain; decimalLatitude: 46.91; decimalLongitude: 47.91; **Event:** samplingProtocol: cow dung washing; eventDate: 2006-10-07; **Record Level:** collectionID: urn:lsid:biocol.org:col:34969; institutionCode: ZIN; collectionCode: Coleoptera
- **Type status:** Other material. **Occurrence:** recordedBy: A. V. Frolov, L. A. Akhmetova; individualCount: 8; **Location:** country: Russia; stateProvince: Astrakhan'; locality: Dosang environs, fixed sands; decimalLatitude: 46.92; decimalLongitude: 47.92; **Event:** samplingProtocol: larvae hand collecting old horse dung; eventDate: 2007-04-17; **Record Level:** collectionID: urn:lsid:biocol.org:col:34969; institutionCode: ZIN; collectionCode: Coleoptera
- **Type status:** Other material. **Occurrence:** recordedBy: A. V. Frolov, L. A. Akhmetova; individualCount: 9; **Location:** country: Russia; stateProvince: Astrakhan'; locality: Dosang environs, left bank of Akhtuba River, floodplain; decimalLatitude: 46.91; decimalLongitude: 47.91; **Event:** samplingProtocol: larvae hand collecting old horse dung; eventDate: 2007-05-17; **Record Level:** collectionID: urn:lsid:biocol.org:col:34969; institutionCode: ZIN; collectionCode: Coleoptera
- **Type status:** Other material. **Occurrence:** recordedBy: A. V. Frolov, L. A. Akhmetova; individualCount: 8; **Location:** country: Russia; stateProvince: Astrakhan'; locality: Dosang environs, left bank of Akhtuba River, floodplain; decimalLatitude: 46.91; decimalLongitude: 47.91; **Event:** samplingProtocol: larvae hand collecting old horse dung; eventDate: 2007-05-18; **Record Level:** collectionID: urn:lsid:biocol.org:col:34969; institutionCode: ZIN; collectionCode: Coleoptera

##### Ecological interactions

###### Feeds on

Adults were found in horse and cow dung, larvae in horse dung.

##### Distribution

Widely distributed in Europe and Asia up to Baikal Lake in the east.

#### Aphodius sturmi

Harold, 1870

##### Materials

- **Type status:** Other material. **Occurrence:** recordedBy: A. V. Frolov, L. A. Akhmetova; individualCount: 1; **Location:** country: Russia; stateProvince: Astrakhan'; locality: Dosang environs, fixed sands; decimalLatitude: 46.92; decimalLongitude: 47.92; **Event:** samplingProtocol: horse dung washing; eventDate: 2006-10-09; **Record Level:** collectionID: urn:lsid:biocol.org:col:34969; institutionCode: ZIN; collectionCode: Coleoptera
- **Type status:** Other material. **Occurrence:** recordedBy: A. V. Frolov, L. A. Akhmetova; individualCount: 7; **Location:** country: Russia; stateProvince: Astrakhan'; locality: Dosang environs, left bank of Akhtuba River, floodplain; decimalLatitude: 46.91; decimalLongitude: 47.91; **Event:** samplingProtocol: larvae hand collecting old horse dung; eventDate: 2007-05-17; **Record Level:** collectionID: urn:lsid:biocol.org:col:34969; institutionCode: ZIN; collectionCode: Coleoptera
- **Type status:** Other material. **Occurrence:** recordedBy: A. V. Frolov, L. A. Akhmetova; individualCount: 8; **Location:** country: Russia; stateProvince: Astrakhan'; locality: Dosang environs, fixed sands; decimalLatitude: 46.92; decimalLongitude: 47.92; **Event:** samplingProtocol: cow dung washing; eventDate: 2012-05-15; **Record Level:** collectionID: urn:lsid:biocol.org:col:34969; institutionCode: ZIN; collectionCode: Coleoptera

##### Ecological interactions

###### Feeds on

Cattle dung, mostly cow dung.

##### Distribution

Transpalearctic species, mostly in steppe and semidesert zone.

#### Aphodius subterraneus

(Linnaeus, 1758)

##### Materials

- **Type status:** Other material. **Occurrence:** recordedBy: A. V. Frolov, L. A. Akhmetova; individualCount: 4; **Location:** country: Russia; stateProvince: Astrakhan'; locality: Dosang environs, fixed sands; decimalLatitude: 46.92; decimalLongitude: 47.92; **Event:** samplingProtocol: cow dung washing; eventDate: 2007-04-17; **Record Level:** collectionID: urn:lsid:biocol.org:col:34969; institutionCode: ZIN; collectionCode: Coleoptera
- **Type status:** Other material. **Occurrence:** recordedBy: A. V. Frolov, L. A. Akhmetova; individualCount: 8; **Location:** country: Russia; stateProvince: Astrakhan'; locality: Dosang environs, right bank of Akhtuba River, floodplain; decimalLatitude: 46.90; decimalLongitude: 47.90; **Event:** samplingProtocol: larvae hand collecting old horse dung; eventDate: 2007-05-24; **Record Level:** collectionID: urn:lsid:biocol.org:col:34969; institutionCode: ZIN; collectionCode: Coleoptera
- **Type status:** Other material. **Occurrence:** recordedBy: A. V. Frolov, L. A. Akhmetova; individualCount: 1; **Location:** country: Russia; stateProvince: Astrakhan'; locality: Dosang environs, left bank of Akhtuba River, floodplain; decimalLatitude: 46.91; decimalLongitude: 47.91; **Event:** samplingProtocol: horse dung washing; eventDate: 2008-05-05; **Record Level:** collectionID: urn:lsid:biocol.org:col:34969; institutionCode: ZIN; collectionCode: Coleoptera

##### Ecological interactions

###### Feeds on

Cattle dung.

##### Distribution

Distributed throughout Palaearctic region (except for northernmost part), introduced in North America.

#### Aphodius sus

(Herbst, 1783)

##### Materials

- **Type status:** Other material. **Occurrence:** recordedBy: A. V. Frolov, L. A. Akhmetova; individualCount: 1; **Location:** country: Russia; stateProvince: Astrakhan'; locality: Dosang environs, left bank of Akhtuba River, floodplain; decimalLatitude: 46.91; decimalLongitude: 47.91; **Event:** samplingProtocol: horse dung washing; eventDate: 2006-10-06; **Record Level:** collectionID: urn:lsid:biocol.org:col:34969; institutionCode: ZIN; collectionCode: Coleoptera
- **Type status:** Other material. **Occurrence:** recordedBy: A. V. Frolov, L. A. Akhmetova; individualCount: 18; **Location:** country: Russia; stateProvince: Astrakhan'; locality: Dosang environs, left bank of Akhtuba River, floodplain; decimalLatitude: 46.91; decimalLongitude: 47.91; **Event:** samplingProtocol: horse dung washing; eventDate: 2006-10-07; **Record Level:** collectionID: urn:lsid:biocol.org:col:34969; institutionCode: ZIN; collectionCode: Coleoptera
- **Type status:** Other material. **Occurrence:** recordedBy: A. V. Frolov, L. A. Akhmetova; individualCount: 6; **Location:** country: Russia; stateProvince: Astrakhan'; locality: S of Dosang, near Akhtuba River floodplain; **Event:** samplingProtocol: larvae hand collecting from soil; eventDate: 2007-05-01; **Record Level:** collectionID: urn:lsid:biocol.org:col:34969; institutionCode: ZIN; collectionCode: Coleoptera

##### Ecological interactions

###### Feeds on

Adults occur in large numbers in horse dung in the fall. Larvae were collected among grass roots at the depth of 10–15 cm in riverine secondary forest (Frolov 2009).

##### Distribution

Middle and southern Europe, Minor Asia, North Africa, northern Iran, Central Asia up to South Siberia.

#### Aphodius testudinarius

(Fabricius, 1775)

##### Materials

- **Type status:** Other material. **Occurrence:** recordedBy: A. V. Frolov, L. A. Akhmetova; individualCount: 4; **Location:** country: Russia; stateProvince: Astrakhan'; locality: Dosang environs, Akhtuba River floodplain; decimalLatitude: 46.91; decimalLongitude: 47.91; **Event:** samplingProtocol: hand collecting from old horse dung; eventDate: 2007-04-10; **Record Level:** collectionID: urn:lsid:biocol.org:col:34969; institutionCode: ZIN; collectionCode: Coleoptera
- **Type status:** Other material. **Occurrence:** recordedBy: A. V. Frolov, L. A. Akhmetova; individualCount: 4; **Location:** country: Russia; stateProvince: Astrakhan'; locality: Dosang environs, fixed sands; decimalLatitude: 46.92; decimalLongitude: 47.92; **Event:** samplingProtocol: hand collecting from old horse dung; eventDate: 2007-04-17; **Record Level:** collectionID: urn:lsid:biocol.org:col:34969; institutionCode: ZIN; collectionCode: Coleoptera
- **Type status:** Other material. **Occurrence:** recordedBy: A. V. Frolov, L. A. Akhmetova; individualCount: 16; **Location:** country: Russia; stateProvince: Astrakhan'; locality: Dosang environs, fixed sands; decimalLatitude: 46.92; decimalLongitude: 47.92; **Event:** samplingProtocol: hand collecting from old horse dung; eventDate: 2007-04-13/15; **Record Level:** collectionID: urn:lsid:biocol.org:col:34969; institutionCode: ZIN; collectionCode: Coleoptera

##### Ecological interactions

###### Feeds on

Adults occur in dry cattle dung.

##### Distribution

Distributed mostly in steppe zone from South Europe to Central Kazakhstan.

#### Aphodius varians

Duftschmidt, 1805

##### Materials

- **Type status:** Other material. **Occurrence:** recordedBy: A. V. Frolov, L. A. Akhmetova; individualCount: 7; **Location:** country: Russia; stateProvince: Astrakhan'; locality: Dosang Railway Station; decimalLatitude: 46.90; decimalLongitude: 47.92; **Event:** samplingProtocol: light trap; eventDate: 2007-05-23; **Record Level:** collectionID: urn:lsid:biocol.org:col:34969; institutionCode: ZIN; collectionCode: Coleoptera
- **Type status:** Other material. **Occurrence:** recordedBy: A. V. Frolov, L. A. Akhmetova; individualCount: 5; **Location:** country: Russia; stateProvince: Astrakhan'; locality: Dosang environs, left bank of Akhtuba River, floodplain; decimalLatitude: 46.91; decimalLongitude: 47.91; **Event:** samplingProtocol: horse dung washing; eventDate: 2008-05-05; **Record Level:** collectionID: urn:lsid:biocol.org:col:34969; institutionCode: ZIN; collectionCode: Coleoptera
- **Type status:** Other material. **Occurrence:** recordedBy: A. V. Frolov, L. A. Akhmetova; individualCount: 1; **Location:** country: Russia; stateProvince: Astrakhan'; locality: Dosang environs, fixed sands; decimalLatitude: 46.92; decimalLongitude: 47.92; **Event:** samplingProtocol: cow dung washing; eventDate: 2012-05-15; **Record Level:** collectionID: urn:lsid:biocol.org:col:34969; institutionCode: ZIN; collectionCode: Coleoptera

##### Ecological interactions

###### Feeds on

Adults feed on cattle dung.

##### Distribution

Widely distributed in western Palaearctic region up to Zaissan Lake in the east.

#### Aphodius variicolor

D. Koshantschikov, 1895

##### Materials

- **Type status:** Other material. **Occurrence:** recordedBy: K. A. Grebennikov; individualCount: 2; **Location:** country: Russia; stateProvince: Astrakhan'; locality: Dosang environs, fixed sands; **Event:** eventDate: 1996-09-13; **Record Level:** collectionID: urn:lsid:biocol.org:col:34969; institutionCode: ZIN; collectionCode: Coleoptera
- **Type status:** Other material. **Occurrence:** recordedBy: A. V. Frolov, L. A. Akhmetova; individualCount: 4; **Location:** country: Russia; stateProvince: Astrakhan'; locality: 15 km NE of Dosang; decimalLatitude: 47.00; decimalLongitude: 47.98; **Event:** samplingProtocol: larvae hand collecting from soil; eventDate: 2008-04-07; **Record Level:** collectionID: urn:lsid:biocol.org:col:34969; institutionCode: ZIN; collectionCode: Coleoptera
- **Type status:** Other material. **Occurrence:** recordedBy: A. V. Frolov; individualCount: 10; **Location:** country: Russia; stateProvince: Astrakhan'; locality: 15 km NE of Dosang; decimalLatitude: 47.00; decimalLongitude: 47.98; **Event:** samplingProtocol: cow dung washing; eventDate: 2010-10-12; **Record Level:** collectionID: urn:lsid:biocol.org:col:34969; institutionCode: ZIN; collectionCode: Coleoptera
- **Type status:** Other material. **Occurrence:** recordedBy: A. V. Frolov, L. A. Akhmetova; individualCount: 1; **Location:** country: Russia; stateProvince: Astrakhan'; locality: Dosang environs, fixed sands; decimalLatitude: 46.92; decimalLongitude: 47.92; **Event:** samplingProtocol: cow dung washing; eventDate: 2012-05-15; **Record Level:** collectionID: urn:lsid:biocol.org:col:34969; institutionCode: ZIN; collectionCode: Coleoptera
- **Type status:** Other material. **Occurrence:** recordedBy: A. V. Frolov, L. A. Akhmetova; individualCount: 3; **Location:** country: Russia; stateProvince: Astrakhan'; locality: Dosang environs, fixed sands; decimalLatitude: 46.92; decimalLongitude: 47.92; **Event:** samplingProtocol: cow dung washing; eventDate: 2006-10-7/11; **Record Level:** collectionID: urn:lsid:biocol.org:col:34969; institutionCode: ZIN; collectionCode: Coleoptera

##### Ecological interactions

###### Feeds on

**Figure 7.**
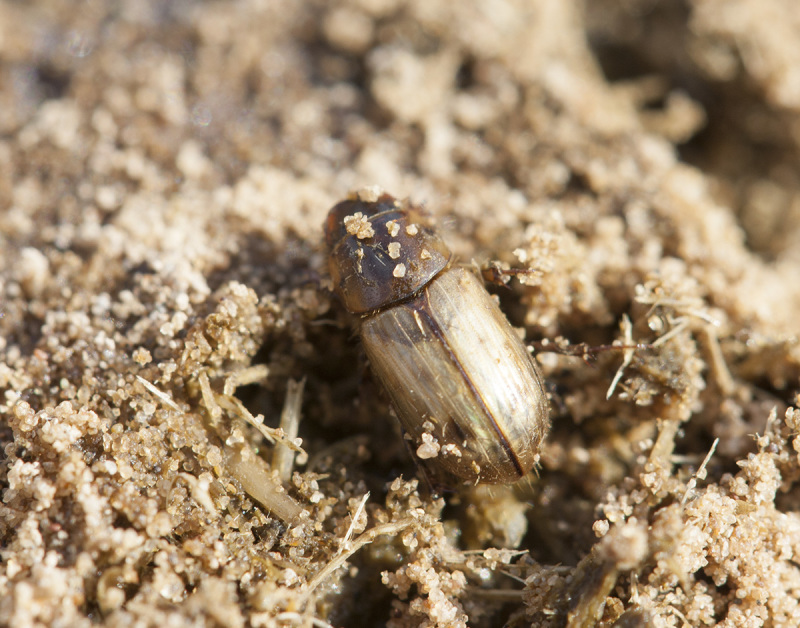
*Aphodius variicolor*. Dosang environs, Astrakhan Province, Russia.

**Figure 8.**
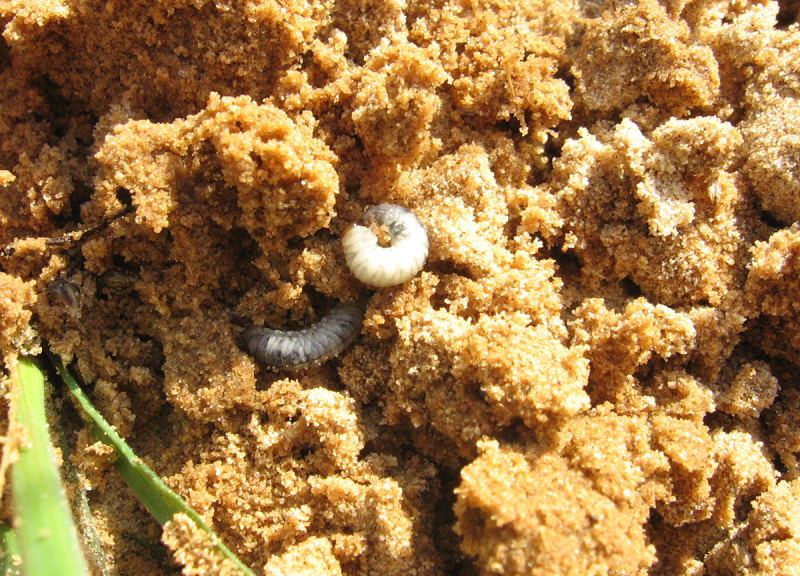
*Aphodius variicolor*, 3-instar larvae. Dosang environs, Astrakhan Province, Russia.

Adults feed on horse dung (Fig. 7), larvae were collected among the roots of cheat grass (Frolov 2009) (Fig. 8).

##### Distribution

Caspian lowland desert.

#### Aphodius zangi

A. Schmidt, 1906

##### Materials

- **Type status:** Other material. **Occurrence:** recordedBy: A. V. Frolov, L. A. Akhmetova; individualCount: 30; **Location:** country: Russia; stateProvince: Astrakhan'; locality: Dosang environs, right bank of Akhtuba River, floodplain; decimalLatitude: 46.90; decimalLongitude: 47.90; **Event:** samplingProtocol: hand collecting from marmot hole; eventDate: 2007-04-22; **Record Level:** collectionID: urn:lsid:biocol.org:col:34969; institutionCode: ZIN; collectionCode: Coleoptera
- **Type status:** Other material. **Occurrence:** recordedBy: A. V. Frolov, L. A. Akhmetova; individualCount: 47; **Location:** country: Russia; stateProvince: Astrakhan'; locality: Dosang environs, right bank of Akhtuba River, floodplain; decimalLatitude: 46.90; decimalLongitude: 47.90; **Event:** samplingProtocol: hand collecting from marmot hole; eventDate: 2008-04-3/9; **Record Level:** collectionID: urn:lsid:biocol.org:col:34969; institutionCode: ZIN; collectionCode: Coleoptera

##### Ecological interactions

###### Feeds on

**Figure 9.**
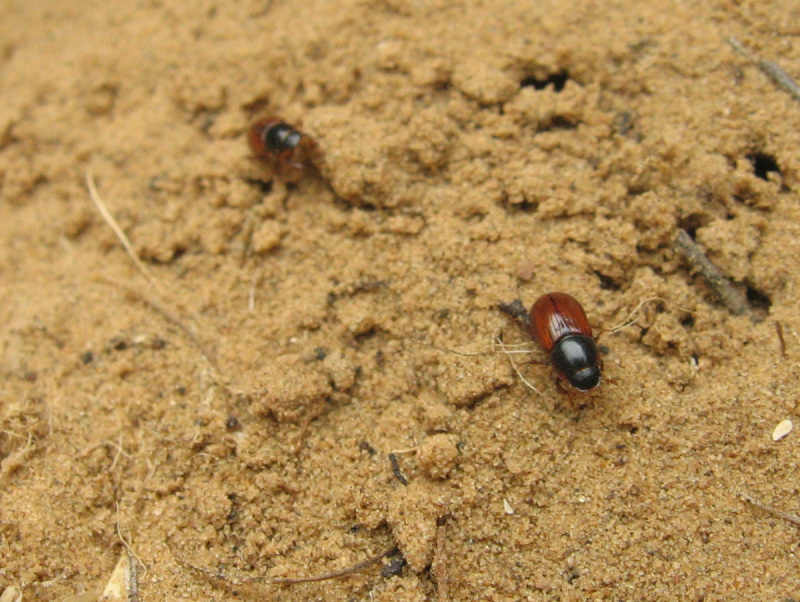
*Aphodius zangi* clambering out of a marmot hole. Dosang environs, Astrakhan Province, Russia.

Rodent nest dweller, adults and apparently larvae feed on rodent excrement (Fig. 9).

##### Distribution

Distributed mostly in the steppe and semidesert zones from Lower Volga in the west to West Siberia in the east.

#### Aphodius vittatus

Say, 1825

##### Materials

- **Type status:** Other material. **Occurrence:** recordedBy: A. V. Frolov, L. A. Akhmetova; individualCount: 15; **Location:** country: Russia; stateProvince: Astrakhan'; locality: Dosang environs, left bank of Akhtuba River, floodplain; decimalLatitude: 46.91; decimalLongitude: 47.91; **Event:** samplingProtocol: cow dung washing; eventDate: 2007-05-21; **Record Level:** collectionID: urn:lsid:biocol.org:col:34969; institutionCode: ZIN; collectionCode: Coleoptera
- **Type status:** Other material. **Occurrence:** recordedBy: A. V. Frolov, L. A. Akhmetova; individualCount: 5; **Location:** country: Russia; stateProvince: Astrakhan'; locality: Dosang environs, fixed sands; decimalLatitude: 46.92; decimalLongitude: 47.92; **Event:** samplingProtocol: cow dung washing; eventDate: 2012-05-15; **Record Level:** collectionID: urn:lsid:biocol.org:col:34969; institutionCode: ZIN; collectionCode: Coleoptera
- **Type status:** Other material. **Occurrence:** recordedBy: A. V. Frolov, L. A. Akhmetova; individualCount: 1; **Location:** country: Russia; stateProvince: Astrakhan'; locality: Dosang environs, fixed sands; decimalLatitude: 46.92; decimalLongitude: 47.92; **Event:** samplingProtocol: sand sifting; eventDate: 2006-10-7/11; **Record Level:** collectionID: urn:lsid:biocol.org:col:34969; institutionCode: ZIN; collectionCode: Coleoptera

##### Ecological interactions

###### Feeds on

Cattle and wild herbivore dung.

##### Distribution

This species is thought to be widely distributed in the Holarctic Region. However, Wilson and Angus (2006) demonstrated that the nominative subspecies, *Aphodius vittatus vittatus* Say, 1825, occurring in North America, has a karyotype different from that of Palearctic subspecies *Aphodius vittatus mundus* Reitter, 1892, *Aphodius vittatus sellatus* Mannerheim, 1852, and *Aphodius vittatus tjanshanicus* Balthasar, 1956. Therefore, *Aphodius vittatus* can be a complex of closely related species. In the Lower Volga region, *Aphodius vittatus mundus* occurs.

#### Cnemisus rufescens

(Motschulsky, 1845)

##### Materials

- **Type status:** Other material. **Occurrence:** recordedBy: A. V. Frolov, L. A. Akhmetova; individualCount: 30; **Location:** country: Russia; stateProvince: Astrakhan'; locality: Dosang environs, right bank of Akhtuba River, floodplain; decimalLatitude: 46.90; decimalLongitude: 47.90; **Event:** samplingProtocol: hand collecting from marmot hole; eventDate: 2007-04-22; **Record Level:** collectionID: urn:lsid:biocol.org:col:34969; institutionCode: ZIN; collectionCode: Coleoptera
- **Type status:** Other material. **Occurrence:** recordedBy: A. V. Frolov, L. A. Akhmetova; individualCount: 47; **Location:** country: Russia; stateProvince: Astrakhan'; locality: Dosang environs, right bank of Akhtuba River, floodplain; decimalLatitude: 46.90; decimalLongitude: 47.90; **Event:** samplingProtocol: hand collecting from marmot hole; eventDate: 2008-04-3/9; **Record Level:** collectionID: urn:lsid:biocol.org:col:34969; institutionCode: ZIN; collectionCode: Coleoptera

##### Distribution

Caspian lowland desert.

## Discussion

To date, 44 species of the Aphodiini have been recorded from Dosang and its environs. This is apparently the richest recorded local Aphodiini fauna in Russia (by “local fauna” here we understand the fauna occurring in a small area and consisting of sympatric and parapatric populations of different species). The large number of the Aphodiini species found in the area can be explained by a combination of the following factors:

1. Long vegetative season with high effective heat sum. The beetles were found active from March to November and it is possible that some species may be active during winter months, especially during mild winters. The climate in the area is arid in general but the high level of groundwater in sands, even in the driest months, provides suitable conditions for psammophilous taxa.
2. Large livestock including horses and cows provide abundant food resources throughout the year. In the hottest months however, much of the fresh dung is consumed by the Scarabaeinae dung-beetles, notably *Scarabaeus typhon* Fischer-Waldheim, and *Gymnopleurus mopsus* (Pallas).
3. The collecting area is situated in the transition belt between two floristic districts: Volga-Akhtuba Floodplain (intrazonal) and Desert (zonal) of Caspian Subprovince of Turanian Province (Laktionov 2010).

Apparently due to the last factor, the fauna consists of the two components of different origin. Intrazonal habitats are suitable for a large number of mesophilous species which constitute the core Dosang Aphodiini fauna. These species are widely distributed in the Palearctic region: *Aphodius bimaculatus*, *Aphodius coenosus*, *Aphodius frater*, *Aphodius ictericus*, *Aphodius kraatzi*, *Aphodius lividus*, *Aphodius lugens*, *Aphodius punctipennis*, *Aphodius quadriguttatus*, *Aphodius* satellitius, *Aphodius serotinus*, *Aphodius testudinarius*, *Aphodius varians*, *Aphodius distinctus*, *Aphodius erraticus*, *Aphodius fimetarius*, *Aphodius granarius*, *Aphodius immundus*, *Aphodius melanostictus*, *Aphodius rufipes*, *Aphodius rufus*, *Aphodius scrofa*, *Aphodius sturmi*, *Aphodius subterraneus*, *Aphodius sus*, and *Aphodius vittatus*.

Another part of the fauna comprises species with the ranges mostly limited to the steppe, semidesert, and desert zones. These are the species with sethian (*Aphodius badenkoi*, *Aphodius clathratus*, *Aphodius digitalis*, *Aphodius hauseri*, *Aphodius kizeritskyi*, *Aphodius kisilkumi*, *Aphodius multiplex*), westscythian-northturanian (*Aphodius caspius*, *Aphodius gregarius*, *Aphodius gresseri*, *Aphodius zangi*, *Aphodius curtulus*), northturanian plane ranges (*Aphodius dosangi*, *Aphodius plustschewskii*, *Aphodius variicolor*, *Cnemisus rufescens*), and 2 species (*Aphodius aequalis* and *Aphodius hydrochaeris*) distributed in Mediterranean and Central Asia. In Russia, most of these species occur only in the Lower Volga region.

## Supplementary Material

XML Treatment for Aphodius aequalis

XML Treatment for Aphodius badenkoi

XML Treatment for Aphodius bimaculatus

XML Treatment for Aphodius caspius

XML Treatment for Aphodius clathratus

XML Treatment for Aphodius coenosus

XML Treatment for Aphodius curtulus

XML Treatment for Aphodius digitalis

XML Treatment for Aphodius distinctus

XML Treatment for Aphodius dosangi

XML Treatment for Aphodius erraticus

XML Treatment for Aphodius fimetarius

XML Treatment for Aphodius frater

XML Treatment for Aphodius granarius

XML Treatment for Aphodius gregarius

XML Treatment for Aphodius gresseri

XML Treatment for Aphodius hauseri

XML Treatment for Aphodius hydrochaeris

XML Treatment for Aphodius ictericus

XML Treatment for Aphodius immundus

XML Treatment for Aphodius kizeritskyi

XML Treatment for Aphodius kisilkumi

XML Treatment for Aphodius kraatzi

XML Treatment for Aphodius lividus

XML Treatment for Aphodius lugens

XML Treatment for Aphodius melanostictus

XML Treatment for Aphodius multiplex

XML Treatment for Aphodius plustschewskii

XML Treatment for Aphodius punctipennis

XML Treatment for Aphodius quadriguttatus

XML Treatment for Aphodius rufipes

XML Treatment for Aphodius satellitius

XML Treatment for Aphodius scrofa

XML Treatment for Aphodius serotinus

XML Treatment for Aphodius sturmi

XML Treatment for Aphodius subterraneus

XML Treatment for Aphodius sus

XML Treatment for Aphodius testudinarius

XML Treatment for Aphodius varians

XML Treatment for Aphodius variicolor

XML Treatment for Aphodius zangi

XML Treatment for Aphodius vittatus

XML Treatment for Cnemisus rufescens

## Supplementary files

### Occurences data for checklist of Aphodiini of Dosang environs

**Authors:** Frolov, A. V., Akhmetova, L. A. **Data type:** occurences Supplementary file with occurences data for cheklist of Aphodiini of Dosang environs (Astrakhan Province, European Russia) mostly collected by A. V. Frolov and L. A. Akhmetova in 2006–2012. **File name:** Species_occurrence-1_v1_DwC_Dosang.xls
